# Long-term clinical outcomes of differentiated thyroid cancer patients with biochemical incomplete response after initial radioiodine therapy, a single-center, retrospective analysis

**DOI:** 10.3389/fendo.2026.1770829

**Published:** 2026-01-28

**Authors:** Congcong Wang, Peihang Han, Guohua Qin, Yutian Li, Xufu Wang

**Affiliations:** 1 Department of Nuclear Medicine, The Affiliated Hospital of Qingdao University, Qingdao, Shandong, China; 2 Department of Radiology, Qingdao Women and Children’s Hospital, Qingdao, Shandong, China

**Keywords:** biochemical incomplete response, clinical outcome, differentiated thyroid cancer, progressive disease, radioiodine therapy, stimulated thyroglobulin

## Abstract

**Background:**

Little is known regarding parameters predicting progressive disease (PD) for differentiated thyroid cancer (DTC) patients exhibiting biochemical incomplete response (BIR) after initial radioiodine (RAI) therapy. The aim of this study was to evaluate the long-term clinical outcomes of BIR patients and to establish the determinants of PD.

**Materials & methods:**

172 DTC patients who were classified as BIR after initial RAI therapy between January 2010 to December 2023 in the Affiliated Hospital of Qingdao University were analyzed. All patients were received only one standardized RAI therapy. At the last follow-up, BIR patients were divided into the PD group and the non-progressive disease (NPD) group. PD was defined as the emergence of a new structural lesion or a ≥25% increase in thyroglobulin level. Univariate and multivariate Cox regression models were employed to identify independent risk factors associated with PD. Meanwhile, progression-free survival (PFS) for BIR patients were also assessed.

**Results:**

After a median follow-up of 48.6 months, 40.1% (69/172) patients experienced PD. AJCC T stage (T1-T3a or T3b-T4; HR:2.073, 95%CI: 1.054-4.076, P = 0.035) and stimulated thyroglobulin (sTg, sTg< 50.0 ng/mL or sTg≥ 50.0 ng/mL; HR: 3.056, 95%CI: 1.655-5.644, P<0.001) were verified to be the independent predictive factors of PD. The median PFS of BIR patients was 64.4 months and the 5-year PFS rate was 60.4%.

**Conclusion:**

sTg≥50.0 ng/mL and T3b-T4 stage are robust, clinically accessible markers identifying PD among BIR patients, warranting intensified surveillance and potentially earlier therapeutic reconsideration.

## 1 Introduction

Differentiated thyroid cancer (DTC) is the most prevalent endocrine malignancy, comprising approximately 90% of all thyroid malignancies, and has demonstrated a sustained rise in global incidence in recent decades (1–3). The management of DTC primarily involves a multimodal approach, including total thyroidectomy, radioiodine (RAI) therapy, and subsequent thyroid-stimulating hormone (TSH) suppression therapy (4–8). Among these, postoperative RAI therapy is pivotal for eradicating residual disease and has been associated with improved disease-specific survival (1, 9, 10).

Dynamic risk stratification (DRS) serves as a key tool for assessing the risk of persistent or recurrent disease in DTC (1, 11). This system classifies patients into four response categories based on their initial therapeutic outcome: excellent response (ER), indeterminate response (IDR), biochemical incomplete response (BIR), and structural incomplete response (SIR) (9). BIR is specifically defined by abnormal suppressed thyroglobulin (sup-Tg) or stimulated thyroglobulin (sTg) levels, or rising anti-Tg antibody (TgAb) titers, in the absence of identifiable structural disease on imaging (2, 9, 11). Patients classified with BIR demonstrate a significantly elevated risk for both structural recurrence and disease persistence (12, 13).

Fortunately, a substantial proportion of patients with an initial BIR attain favorable long-term outcomes. Spontaneous reclassification to no evidence of disease status occurs in 33.7% to 38.0% of these patients without further intervention (14, 15). However, the clinical course is heterogeneous; while approximately one-third experience remission, an estimated 17–20% will progress to structurally identifiable disease, necessitating further intervention (13, 16). The specific risk factors predictive of disease progression within the BIR population have not been definitively established. Therefore, this study was designed to evaluate the clinical outcomes and to identify predictors of progressive disease (PD) in patients who exhibited a BIR following initial RAI therapy.

## 2 Materials and methods

### 2.1 Patients

This retrospective study was conducted in the Department of Nuclear Medicine at the Affiliated Hospital of Qingdao University. The study protocol was approved by the Institutional Review Board, with a waiver of informed consent due to its retrospective design and use of anonymized data, in compliance with the Declaration of Helsinki (Ethics approval number: QYFY WZLL 30822). Subject eligibility was determined using predefined inclusion and exclusion criteria, as detailed in **Table 1**.

**Table 1:** Inclusion and exclusion criteria in this study.

Inclusion criteria	Exclusion criteria
• Adults (age ≥18 years) • Total thyroidectomy with selective cervical lymph node dissection for DTC • Intermediate- or high-risk of recurrence according to 2025 ATA guidelines (9) • Received only one standardized RAI therapy between Jan 2010 and Dec 2023 • Initial therapeutic response assessed as BIR	• Age <18 years • Presence of interfering anti-thyroglobulin antibodies (TgAb >115 IU/mL) • History of concomitant malignancy • Receipt of other anti-tumor therapies (e.g., repeated RAI therapy, external beam radiation, targeted therapy, chemotherapy) prior to progression assessment • Inadequate clinical or laboratory data for analysis

DTC, differentiated thyroid carcinoma; ATA, American Thyroid Association; RAI, radioiodine therapy; BIR, biochemical incomplete response; TgAb, anti-thyroglobulin antibody.

### 2.2 Collection of main epidemiological and clinicopathological characteristics

The main epidemiological and clinicopathological parameters were obtained for each patient: gender, age, histologic subtype, multicentricity, tumor size, BRAF^V600E^ mutation status, T-stage, lymph node metastasis, initial recurrence risk stratification, serum sTg level, and TgAb status. All patients were re-staged according to the American Joint Committee on Cancer/Union for International Cancer Control (AJCC/UICC) TNM staging system (8th edition) (17).

### 2.3 RAI protocol and follow-up

The preparation for RAI therapy in DTC patients was conducted in accordance with the 2025 American Thyroid Association (ATA) management guidelines (9). Prior to RAI administration, all patients were placed on a strict low-iodine diet. L-thyroxine treatment was withdrawn for a period of 3–4 weeks to achieve endogenous TSH stimulation, with therapy proceeding only after serum TSH levels exceeded 30 mIU/L. sTg, TgAb levels, along with radiological assessments (neck ultrasonography, chest CT, and, when clinically indicated, MRI or ^18^F-FDG PET/CT) were measured before RAI administration. The administered RAI activity was determined based on individual disease status, ranging from 100 to 150 mCi (13). A post-therapy whole-body scan (Rx-WBS) was acquired 3–7 days following RAI administration (18). Subsequent management included initiation of TSH-suppressive levothyroxine therapy and maintenance of a low-iodine diet for an additional two weeks. Follow-up protocol consisted of an initial assessment at one month to monitor the adequacy of TSH suppression, followed by regular evaluations at 3 to 6 month intervals. These evaluations included serial measurements of TSH, Tg, and TgAb, and were supplemented by neck ultrasonography or chest CT as clinically warranted (12).

### 2.4 Definitions of clinical outcomes

The therapeutic response to initial RAI therapy was assessed at 6–12 months after therapy based on sTg or sup-Tg levels and diagnostic imaging findings, following the 2025 ATA guidelines: (1) excellent response (ER): negative imaging with sTg <1 ng/mL or sup-Tg <0.2 ng/mL; (2) indeterminate response (IDR): nonspecific imaging findings or faint thyroid-bed RAI uptake, accompanied by sTg <10 ng/mL or 0.2 ng/mL≤ sup-Tg< 1 ng/mL; (3) BIR: negative imaging but elevated tumor marker levels (sTg ≥10 ng/mL or sup-Tg ≥1 ng/mL); (4) structural incomplete response (SIR), defined by structural or functional evidence of disease on imaging findings (9).

At final follow-up, therapeutic responses were categorized as ER, IDR, BIR, or SIR. Progressive disease (PD) was defined as either biochemical progression [an increase in sup-Tg of ≥25% from baseline (19, 20)] or structural/functional disease recurrence (e.g., new lesions on imaging), regardless of Tg changes. Thus, patients exhibiting biochemical progression or structural/functional recurrence were classified into the PD group. All other patients, including those with ER, IDR, or BIR without biochemical progression, were assigned to the non-progressive disease (NPD) group. Progression-free survival (PFS) was calculated as the time from initial RAI administration to either biochemical progression or structural/functional disease recurrence (21).

### 2.5 Statistical analysis

Continuous variables are presented as medians and ranges, whereas categorical variables as frequencies and percentages. Between-group comparisons of sTg levels were performed using the Mann-Whitney U test. The optimal cutoff value for sTg in predicting PD was determined by receiver operating characteristic (ROC) curve analysis. PFS was estimated using the Kaplan-Meier method, and survival curves were compared with the log-rank test. Univariable and multivariable Cox regression models were employed to identify independent risk factors associated with PD. All tests were two-sided, and a P value <0.05 was considered statistically significant.

## 3 Results

### 3.1 Baseline characteristics

From January 2010 to December 2023, a total of 172 BIR patients with intermediate- or high-risk were included in this study. The baseline characteristics were reported in **Table 2**. Papillary thyroid carcinoma (PTC) was diagnosed in 167 patients (97.1%), and follicular thyroid carcinoma (FTC) was diagnosed in 5 patients (2.9%). The primary tumor size was 2.0 (1.2-3.0) cm. Among these, 65.7% (113/172) were classified as AJCC T1-T3a, yet merely 34.3% (59/172) patients were classified as T3b -T4, respectively. Regarding N staging, 127 patients (73.8%) were classified as N1b. In addition, multicentricity was observed in 100 patients (58.1%), and the BRAF^V600E^ mutations positive was detected in 78 patients (45.3%). According to the 2025 ATA risk stratification, 67.4% (116/172) patients were intermediate-risk, and 32.6% (56/172) patients were high-risk. The sTg levels ranged from 13.0 to 305.0 ng/mL, with a median of 47.2 ng/mL. Administered RAI activities varied from 100 to 150 mCi.

**Table 2:** Clinicopathologic characteristics of BIR patients (n=172).

Characteristics	Patients n(%)	Median (IQR)	Range
Age (years)		39.5 (31, 55)	18-68
<55	128 (74.4)		
≥55	44 (25.6)		
Gender			
Male	70 (40.7)		
Female	102 (59.3)		
Histological type			
PTC	167 (97.1)		
FTC	5 (2.9)		
Multicentricity			
Yes	100 (58.1)		
No	72 (41.9)		
Tumor size (cm)		2.0 (1.2, 3.0)	0.3-6.0
T stage			
T1-T3a	113 (65.7)		
T3b-T4	59 (34.3)		
N stage			
N0-N1a	45 (26.2)		
N1b	127 (73.8)		
BRAF^V600E^ mutations			
NO/NA	94 (54.7%)		
Yes	78 (45.3%)		
Recurrence risk			
Intermediate	116 (67.4%)		
High	56 (32.6%)		
sTg (ng/mL)		47.2 (27.9, 79.1)	13.0-305.0
RAI activity (mCi)			100-150
100	34 (19.8)		
120	24 (14)		
125	3 (1.7)		
150	111 (64.5)		
Follow-up duration (months)		48.6 (42.9, 63.1)	10.3-103.4

BIR, biochemical incomplete response; RAI, radioiodine therapy; PTC, papillary thyroid cancer; FTC, follicular thyroid cancer; NA, Not applicable; T, tumor; N, node; sTg, stimulated thyroglobulin; RAI, radioiodine.

### 3.2 Clinical outcomes of BIR patients

**Figure 1:**
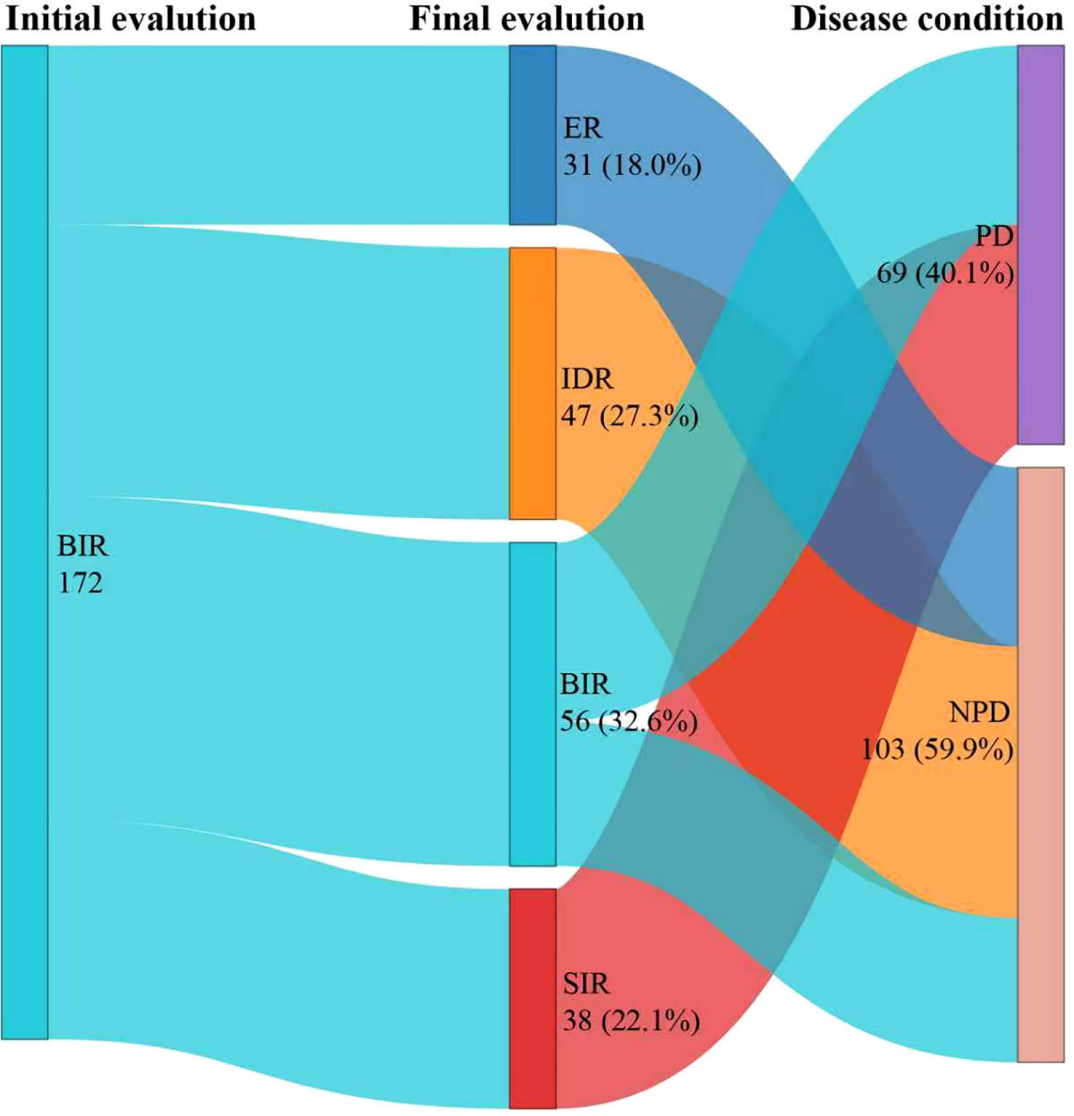
This Sankey diagram illustrates the dynamic shifts in disease status among BIR patients between initial and final follow-up evaluations. All patients underwent only one RAI therapy. The therapeutic response to initial RAI was evaluated after 6–12 months. ER, excellent response; IDR, indeterminate response; BIR, biochemical incomplete response; SIR, structural incomplete response; PD, progressive disease; NPD, non-progressive disease; RAI, radioiodine therapy.

After a median follow-up of 48.6 months (IQR, 42.9-63.1 months), **Figure 1** presented a Sankey diagram illustrating the dynamic shifts in disease status among BIR patients between initial and final follow-up evaluations. By the final follow-up, 56 (32.6%) patients were classified as having a BIR, 38 (22.1%) patients as SIR, 47 (27.3%) patients as IDR, and 31 (18.0%) patients as ER. Notably, the majority of patients (103, 59.9%) had attained NPD, whereas 69 (40.1%) exhibited PD, indicative of either structural persistence or biochemical progression.

### 3.3 Predictive value of sTg in BIR patients with PD

**Figure 2:**
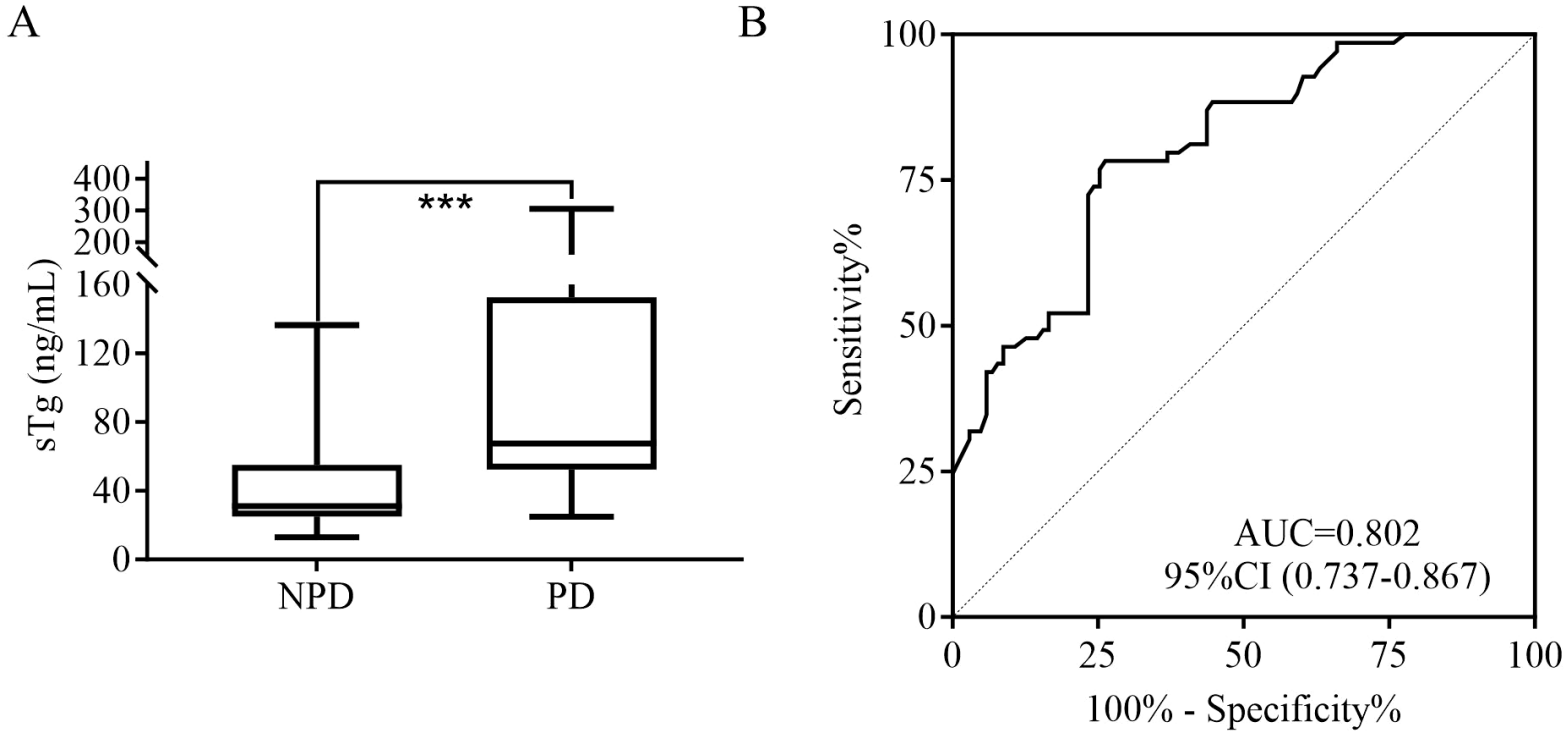
**(A)** Comparison of the sTg level for PD and NPD in BIR patients. **(B)** ROC curves of sTg for detecting PD in BIR patients. sTg, stimulated thyroglobulin; PD, progressive disease; NPD, non-progressive disease; BIR, biochemical incomplete response; RAI, radioiodine therapy; ROC, receiver operating characteristic; AUC, area under the ROC curve; ***P<0.001.

The sTg level in the PD group was 67.6 (52.4, 152.6) ng/mL, which was significantly higher than the 31.0 (25.1, 55.1) ng/mL observed in the NPD group (**Figure 2A**). ROC curve analysis for BIR patients (**Figure 2B**) identified the optimal sTg threshold of 50.0 ng/mL for predicting PD, with corresponding sensitivity of 78.3%, specificity of 73.8% and an AUC of 0.774, respectively.

### 3.4 PFS of BIR patients

**Figure 3:**
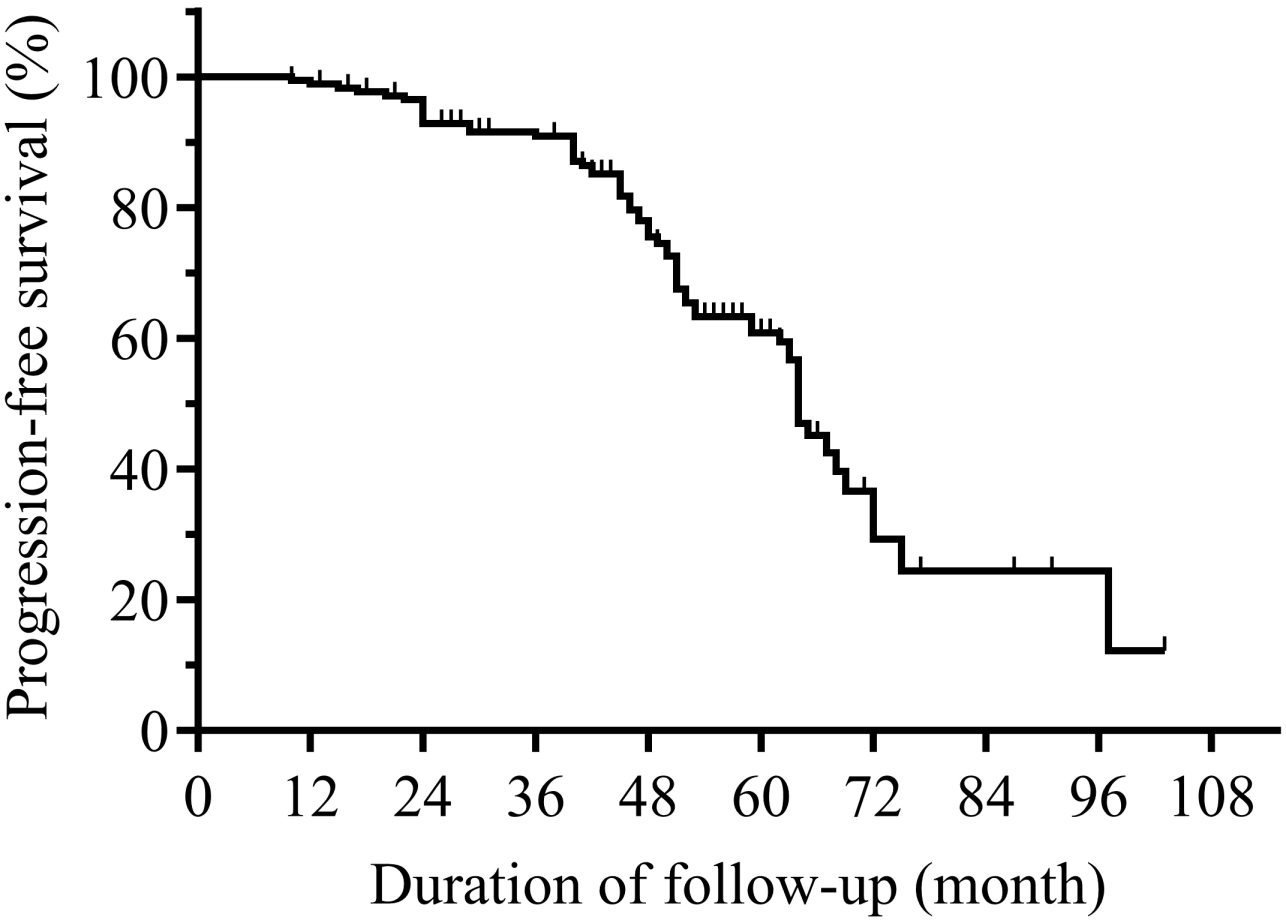
Kaplan-Meier progression-free survival (PFS) curves for BIR patients. BIR, biochemical incomplete response.

**Figure 4:**
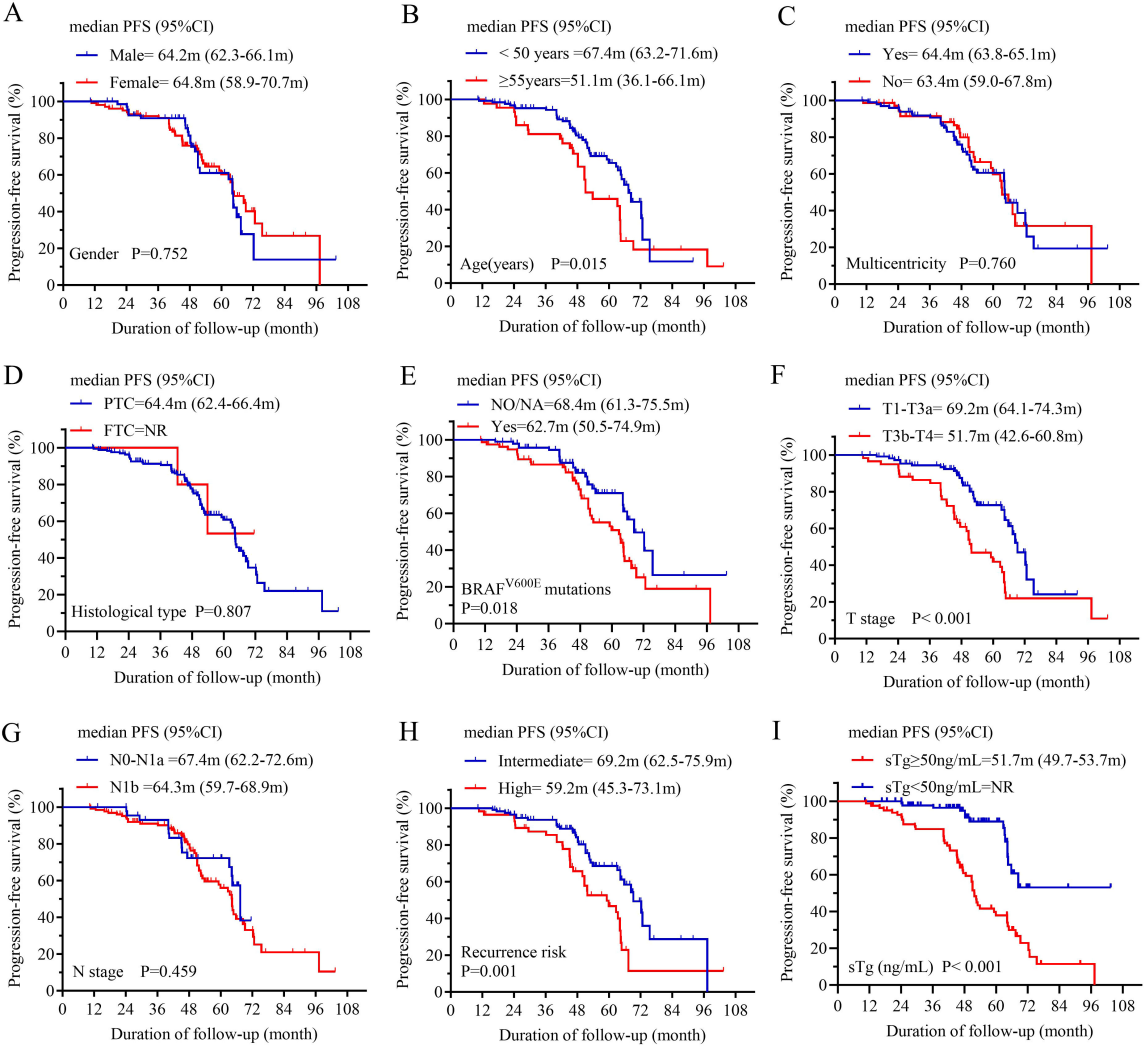
Comparison of progression-free survival (PFS) curves for BIR patients according to **(A)** gender, **(B)** age, **(C)** multicentricity, **(D)** histological type, **(E)** BRAF^V600E^ mutations, **(F)** T stage, **(G)** N stage, **(H)** recurrence risk, and **(I)** sTg. BIR, biochemical incomplete response; T, tumor; N, node; NA, Not applicable; sTg, stimulated thyroglobulin; NR, not reached.

In the analysis of PFS, 69 patients had disease progression. The median PFS of BIR patients was 64.4 months (**Figure 3**). Then, the 1-year, 3-year, 5-year, and 6-year PFS rates of BIR patients were 99.4%, 91.5%, 60.4%, and 35.5%, respectively. Moreover, subgroup analysis revealed significant differences between the subgroups in terms of the age (**Figure 4B**), BRAF^V600E^ mutations (**Figure 4E**), T stage (**Figure 4F**), recurrence risk (**Figure 4H**) and sTg (**Figure 4I**), while there were no significant differences between subgroups based on gender (**Figure 4A**), multicentricity (**Figure 4C**), histological type (**Figure 4D**), or N stage (**Figure 4G**).

### 3.5 Influence factors, univariable and multivariable Cox regression

Factors including gender (female or male), age (<55 years or ≥55years), tumor size, histological type (PTC or FTC), multicentricity (Yes or No), AJCC T stage (T1-T3a or T3b-T4), N stage (N0-N1a or N1b), BRAF^V600E^ mutations (NO/NA or Yes), recurrence risk (intermediate or high), RAI activity (100 mCi, 120 mCi, 125 mCi or 150 mCi) and sTg (< 50.0 ng/mL or ≥ 50.0 ng/mL), were analyzed using the univariate and multivariate Cox regression. In univariate Cox regression analyses, the age (hazard ratio (HR): 1.839, 95% confidence interval (CI): 1.120-3.020, P = 0.016), BRAF^V600E^ mutations (HR:1.776, 95%CI: 1.094–2.883, P = 0.020), T stage (HR:2.606, 95%CI: 1.613-4.210, P<0.001), recurrence risk (HR: 2.270, 95%CI: 1.404 - 3.672, P<0.001)) and sTg (HR: 4.056, 95%CI: 2.286-7.198, P<0.001) were eligible to predict PD. In multivariate Cox regression analyses, only the T stage (HR:2.073, 95%CI: 1.054 - 4.076, P = 0.035) and sTg (HR: 3.056, 95%CI: 1.655-5.644, P<0.001) were verified to be the independent predictive factors in detecting PD (**Table 3**).

**Table 3:** Cox regression analyses of the baseline factors and PD.

Characteristics	Total(N)	Univariate analysis		Multivariate analysis	
		Hazard ratio (95% CI)	P value	Hazard ratio (95% CI)	P value
Gender	172				
Female	102	Reference		Reference	
Male	70	1.083 (0.664 - 1.766)	0.749	0.852 (0.499 - 1.453)	0.556
Age (years)	172				
<55	128	Reference		Reference	
≥55	44	1.839 (1.120 - 3.020)	**0.016**	1.144 (0.678 - 1.930)	0.613
Histological type	172				
PTC	167	Reference		Reference	
FTC	5	0.837 (0.204 - 3.427)	0.805	1.086 (0.258 - 4.571)	0.910
Multicentricity	172				
No	72	Reference		Reference	
Yes	100	1.080 (0.663 - 1.760)	0.756	1.127 (0.652 - 1.948)	0.669
Tumor size (cm)	172	1.149 (0.948 - 1.392)	0.156	0.845 (0.640 - 1.116)	0.236
BRAF^V600E^ mutations	172				
NO/NA	94	Reference		Reference	
Yes	78	1.776 (1.094 - 2.883)	**0.020**	1.125 (0.673 - 1.882)	0.653
T stage	172				
T1-3a	113	Reference		Reference	
T3b-T4	59	2.606 (1.613 - 4.210)	**< 0.001**	2.073 (1.054 - 4.076)	**0.035**
N stage	172				
N1b	127	Reference		Reference	
N0-1a	45	0.803 (0.450 - 1.435)	0.459	1.249 (0.646 - 2.418)	0.508
Recurrence risk	172				
Intermediate	116	Reference		Reference	
High	56	2.270 (1.404 - 3.672)	**< 0.001**	1.088 (0.550 - 2.154)	0.808
RAI activity (mCi)	172				
150	111	Reference		Reference	
120	24	0.280 (0.100 - 0.780)	**0.015**	0.471 (0.155 - 1.434)	0.185
100	34	0.808 (0.460 - 1.418)	0.457	0.908 (0.511 - 1.615)	0.743
125	3	0.000 (0.000 - Inf)	0.997	0.000 (0.000 - Inf)	0.997
sTg (ng/mL)	172				
<50.0	91	Reference		Reference	
≥50.0	81	4.056 (2.286 - 7.198)	**< 0.001**	3.056 (1.655 - 5.644)	**< 0.001**

BIR, biochemical incomplete response; PTC, papillary thyroid cancer; FTC, follicular thyroid cancer; NA, Not applicable; T, tumor; N, node; sTg, stimulated thyroglobulin; RAI, radioiodine. The bold text indicates the observation indicators that are of particular significance and require emphasis.

## 4 Discussion

RAI therapy represents a cornerstone in the postoperative management of DTC, aimed at eradicating residual disease and reducing recurrence risk (8, 9). Despite the generally favorable prognosis associated with DTC, a notable subset of patients, approximately 11–22%, exhibit a BIR following initial RAI therapy (2, 22). Among these BIR patients, an estimated 17–20% will eventually progress to structurally identifiable disease, underscoring the clinical challenge of risk stratification within this heterogeneous population (13, 16).

In a retrospective analysis by Ahn et al. (13), longitudinal surveillance outcomes were evaluated in a cohort of 266 BIR patients who were managed without additional RAI intervention. Unfortunately, after a median follow-up of 12 years, structural recurrence was identified in 43 patients, representing a substantial proportion of the cohort. In the present study, our findings reveal a distinct prognostic dichotomy: while a majority (59.9%) maintained NPD status over a median follow-up of 48.6 months, a substantial proportion (40.1%) experienced disease progression. The 5-year PFS rate of BIR patients was 60.4%. Additionally, the clinical factors associated with PFS in BIR cohort were assessed, and it was observed that patients with older age at diagnosis, presence of the BRAF^V600E^ mutation, advanced tumor staging (T3b-T4), high recurrence risk stratification, and sTg levels≥50.0 ng/mL might have a poorer PFS than other patients. However, other clinical factors such as gender, multifocality, histological type, and N stage were not associated with PFS. Multivariate Cox regression analysis identified sTg ≥50.0 ng/mL and advanced primary tumor stage (T3b–T4) as independent predictors of PD. These results highlight the necessity of refining risk stratification beyond the broad BIR classification by incorporating readily available clinicopathological and biochemical markers, thereby facilitating individualized surveillance and informing timely therapeutic intervention.

The observed PD rate of 40.1% aligns with and substantiates the non-indolent nature of a significant BIR subset reported in prior literature (22, 23), reinforcing that biochemical persistence signifies active, albeit radiologically occult, disease burden in many patients. Conversely, the 18.0% rate of spontaneous reclassification to an ER status without additional RAI therapy validates the current conservative, observative approach for a portion of BIR patients, as recommended by 2025 ATA paradigms (9).

Indeed, spontaneous decline in Tg levels, ultimately leading to reclassification as remission without any additional therapy, has been documented in 6–33% of BIR patients (2, 13, 14, 24). The likelihood of this spontaneous biochemical remission is inversely correlated with the initial sTg level. This relationship was quantified by Vaisman et al. (14) in a cohort of 77 BIR patients, where the median sTg was significantly lower in those who achieved spontaneous remission (13 ng/mL) compared to those who did not (33 ng/mL; p = 0.03). Notably, in a retrospective study of 32 BIR patients after initial RAI therapy, Pitoia et al. (25) reported that after a median follow-up of 6 years, no patient with a sTg level >10 ng/mL transitioned spontaneously to remission status. Our data suggest that the sTg level serves as a potent surrogate for residual tumor volume and biologic aggressiveness. The strong predictive performance of the 50.0 ng/mL cutoff (AUC 0.774) provides a quantitative, clinically actionable threshold. sTg levels ≥50.0 ng/mL likely reflect a higher tumor burden not yet detectable by conventional anatomic imaging, portending a greater likelihood of eventual biochemical or structural progression. This finding is physiologically coherent, as Tg secretion correlates with functional thyroid tissue mass, and its persistence after thyroidectomy and RAI ablation indicates incomplete response (9, 26, 27).

The independent prognostic value of advanced T stage (T3b-T4) in our multivariate analysis, is particularly noteworthy. It underscores that the intrinsic locoregional invasiveness of the primary tumor, specifically gross extrathyroidal extension. This observation is corroborated by extant literature, which consistently identifies advanced T staging, as a predictor of diminished therapeutic response to RAI therapy (28, 29). The underlying pathophysiological basis for this association lies in the aggressive tumor biology and extensive local invasion characteristic of T3b-T4 lesions, factors which are known to compromise radioiodine avidity and efficacy. These findings are further supported by quantitative analyses, such as that by Wang et al. (30), which established a significant correlation between T4 staging and inferior survival metrics, including overall survival and disease-specific survival. The adverse impact of advanced T stage on prognosis is a consistent theme, as similarly demonstrated by Li et al. (31) in a cohort of stage IVB DTC patients. Clinically, the T4 stage in advanced DTC signifies a greater initial tumor burden and a more invasive phenotype, which collectively contribute to a higher risk of structural recurrence and poorer long-term outcomes. Consequently, these data collectively underscore the clinical imperative to consider more vigilant surveillance and the potential need for personalized, multi-modal therapeutic strategies in this high-risk patient subgroup.

Interestingly, while age, BRAF^V600E^ mutation status and initial ATA recurrence risk stratification (high vs. intermediate) were significant predictors on univariate analysis, they did not retain independence in the multivariate model. This finding, particularly for BRAF^V600E^, can be interpreted in the context of established tumor biology and statistical mediation. The BRAF^V600E^ mutation is a well-documented driver of aggressive tumor behavior, including local invasion and metastatic potential (32, 33). While its significant univariate association with PD, its loss of independence in the multivariable model suggests that its prognostic effect is largely mediated by its correlation with more advanced T stage and higher sTg levels, which themselves emerged as dominant predictors. This suggests that within the BIR population, the anatomic extent of disease (T stage) and the quantitative biochemical burden (sTg) serve as more proximate and powerful integrators of risk, potentially capturing the downstream clinical consequences of the BRAF^V600E^ mutation. Furthermore, the sample size of our cohort may have been limited in detecting a modest independent effect of BRAF^V600E^ status after adjusting for these strong predictors. Therefore, while BRAF^V600E^ remains an important biological marker, its utility for independent risk stratification in BIR patients may be superseded by the combined assessment of T stage and sTg.

From a clinical nuclear medicine perspective, these findings have direct implications for practice. Patients presenting with BIR and either sTg ≥50.0 ng/mL or a primary T3b-T4 tumor constitute a high-risk subgroup warranting intensified surveillance. This could entail more frequent serial assessments of serum biomarkers (specifically Tg and TgAb), alongside anatomical imaging such as neck ultrasonography and chest CT. Furthermore, a lower threshold for employing advanced functional imaging such as 18F-FDG PET/CT to detect occult metastatic disease. In cases where clinical or biochemical suspicion persists, the feasibility of empiric repeat RAI therapy may warrant earlier discussion, particularly if there is a reasonable expectation of residual RAI-avid tissue. Conversely, BIR patients with sTg <50.0 ng/mL and lower T stage may be reasonably managed with standard-interval follow-up, avoiding unnecessary interventions given their higher probability of stable disease or spontaneous remission.

Several limitations of this study must be acknowledged. Its retrospective, single-center design inherently carries risks of selection and information bias. The exclusion of patients with TgAb positive, while methodologically necessary for accurate sTg interpretation, limits the generalizability of the sTg threshold to the approximately 20-25% of DTC patients with interfering antibodies. The administered RAI activities (100–150 mCi) followed a standardized protocol, but the lack of dosimetric assessment precludes correlation between delivered radiation dose to metastases and biochemical response. Furthermore, the median follow-up of just over 4 years, while informative, is still relatively short for a typically indolent malignancy like DTC; longer-term data are needed to confirm the stability of these predictive relationships and final disease-specific survival outcomes.

## 5 Conclusion

In conclusion, DTC patients exhibiting BIR after initial RAI therapy had a high risk of biochemical progression or structural disease recurrence and only a small proportion of patients reverted to ER after long-term follow-up. We propose that sTg ≥50.0 ng/mL and T3b-T4 disease be considered high-risk features within the BIR classification, prompting a more vigilant management pathway. These readily available parameters enhance the granularity of dynamic risk stratification, facilitating personalized follow-up strategies and timely therapeutic decisions.

## Data Availability

The original contributions presented in the study are included in the article/supplementary material. Further inquiries can be directed to the corresponding author.
